# Interpenetrating Polymer Networks Based on Bacterial Cellulose and Poly(acrylic acid–co-N, N-methylene-bis-acrylamide) as Carriers for Phytoextracts

**DOI:** 10.3390/gels12070624

**Published:** 2026-07-11

**Authors:** Anamaria Zaharia, Anita-Laura Chiriac, Marinela-Victoria Iordanescu, Bianca Elena Stoica, Andrei Sarbu, Tanta-Verona Iordache

**Affiliations:** Advanced Polymer Materials and Polymer Recycling Group, National Institute for Research & Development in Chemistry and Petrochemistry ICECHIM, Spl. Independentei 202, 6th District, 060021 Bucharest, Romania; anamaria.zaharia@icechim.ro (A.Z.); anita-laura.radu@icechim.ro (A.-L.C.); marinela.dumitru@icechim.ro (M.-V.I.); bianca-elena.stoica@icechim.ro (B.E.S.)

**Keywords:** interpenetrating polymer networks, hydrogels, carriers, phytoextract, sustainable agricultural solutions

## Abstract

Climate change and population growth are intensifying global food security challenges by reducing agricultural productivity and increasing reliance on fertilizers. In this context, developing sustainable and economically efficient agricultural solutions becomes essential. The study presents the synthesis of an interpenetrating polymer network (IPN) of hydrogels by combining bacterial cellulose (BC) with poly(acrylic acid) crosslinked with N, N-methylene-bis-acrylamide (PAA–co–MBA) via free radical copolymerization. To explore their potential as bioactive compound carriers, an ethanolic hydroalcoholic phytoextract (EHP) obtained from *Hypericum perforatum* L. and *Melissa officinalis* L. was directly encapsulated within the IPN hydrogels. The EHP is valued for its rich bioactive profile and antifungal, antimycobacterial, and antioxidant properties. The results of rheology measurements and thermal gravimetric analysis (TGA) revealed that incorporating BC into the IPN hydrogels significantly enhanced the mechanical stiffness, thermal resistance, and overall stability of the resulting IPN structures. Fourier Transform Infrared (FTIR) spectroscopy and Scanning Electron Microscopy (SEM) confirmed the structural organization and the porosity of the developed composite, as well as the successful fabrication of IPN hydrogels in the EHP medium. Under optimal conditions, the IPN hydrogels exhibited a reduced swelling capacity, thereby slowing the diffusion of the bioactive agents, reducing the application frequency, and enhancing the utilization efficiency. Taken together with the controlled-release performance, these findings demonstrate the potential of BC (PAA-co-MBA) IPN hydrogels as biodegradable and sustainable carrier systems for controlled delivery applications and suggest that they may be promising candidates for hydrogel-based agricultural delivery systems.

## 1. Introduction

Conventional agriculture has significantly contributed to meeting the nutritional demands of an expanding global population. However, this progress has been accompanied by the increased use of synthetic fertilizers and pesticides, leading to soil degradation and a decline in overall environmental quality [1]. Growing awareness of the ecological risks associated with these practices has raised concerns regarding the long-term sustainability of modern agricultural systems.

Hydrogels are three-dimensional hydrophilic polymer networks characterized by a high water-absorption capacity and adjustable physicochemical properties, making them promising materials for future applications. They are typically formed through the crosslinking of polymer chains via chemical and/or physical interactions [2,3,4]. An interpenetrating polymer network (IPN) consists of two or more polymer networks that are completely or partially interlaced without covalent bonding between them [5]. Owing to their stability, biodegradability, low toxicity, and responsive swelling behavior, they are particularly well-suited for agricultural use [6]. In such systems, hydrogels act as both soil-conditioning agents and controlled-release carriers for nutrients and agrochemicals, improving water availability, nutrient-use efficiency, and supporting plant protection strategies [7,8,9,10,11,12,13,14]. By enhancing soil moisture retention, they promote seed germination and early plant development, while also improving soil quality and increasing resilience under adverse environmental conditions.

Among the hydrogel systems investigated, PAA–co–MBA networks synthesized via free radical copolymerization have received considerable attention. Their high hydrophilicity, pH-responsive swelling, and mechanical stability make them suitable for water retention enhancement, soil conditioning, and controlled nutrient delivery. Studies have shown that PAA-based hydrogels can improve soil moisture dynamics and plant growth under water-limited conditions [15,16]. Other research has incorporated fertilizers, micronutrients, or pesticides into PAA matrices to achieve slow, sustained release, reducing nutrient leaching and increasing fertilizer use efficiency [17,18]. Despite these advances, current studies often focus on swelling behavior or release under specific conditions, without systematically linking synthesis parameters, network structure, and release mechanisms.

In recent years, increasing attention has been directed toward promising natural alternatives, such as plant-derived extracts, which have demonstrated ready availability, cost-effectiveness, and greater environmental compatibility than conventional agrochemicals [19,20]. Beyond their role as food sources, plants contain a wide range of bioactive compounds [21] exhibiting insecticidal properties, antifeedant activity, including repellency, interference with insect growth and development, antimicrobial effects, and the potential to enhance soil fertility [22,23]. The use of plant-based materials, including extracts and isolated phytochemicals, therefore represents a promising strategy for improving soil nutrient profiles in an environmentally sustainable manner [24]. Concentrated plant extracts incorporated into hydrogel matrices enhance their stability, solubility, and bioavailability while allowing for the controlled, sustained release of the active compounds, representing an eco-friendly approach to modern agriculture [25,26,27].

Various methods for incorporating plant extracts within hydrogels can be employed, including in situ polymerization [28], emulsion solvent evaporation [29], microencapsulation [30], nanoprecipitation [31], and ionic gelation [32,33]. Many investigations have focused on synthesizing IPN hydrogels based on biopolymers and a synthetic polymer as carriers for herbal extracts; these hydrogels have mostly been applied in unrelated agricultural applications [34]. For example, Pelin M.I. et al. [35] investigated the encapsulation of hydroalcoholic extract from *Calendula officinalis* into pullulan/poly(vinyl alcohol) hydrogels using an eco-friendly approach that combined covalent and physical crosslinking, aiming to develop materials for wound-healing applications. Similarly, Kedzierska et al. [36] synthesized hydrogel materials based on poly(vinyl alcohol) and polyvinylpyrrolidone, and incorporated wild rose extract into their structure using photopolymerization. The resulting hydrogels were designed as multifunctional wound dressings capable of delivering antioxidant compounds.

In the agricultural field, Clemente I. et al. [13] designed hydrogel beads based on sodium alginate and sodium carboxymethylcellulose for the encapsulation and controlled release of glycoalkaloids extracted from tomato and potato leaves, which are promising biocompatible disinfectants for agricultural soils. In another study, Cadena et al. [26] combined the properties of *Cinnamomum zeylanicum* bark essential oil, alginate, and mesoporous silica nanoparticles (MSNPs) to develop a seed treatment against bacterial phytopathogens. Their system enabled the efficient use of volatile biocides, such as essential oils, at very low concentrations for the prevention and treatment of microbial crop diseases. Furthermore, Li et al. designed a bio-based supramolecular hydrogel composed of tannic acid and quaternized chitosan using a simple shaking method. The resulting material exhibited reversible gel–sol transition behavior and was proposed as a strategy for improving pesticide utilization efficiency in a comprehensive manner [37].

To address these gaps, the present study provides a comprehensive evaluation of PAA–co–MBA hydrogels synthesized under controlled free radical copolymerization conditions, focusing on their structural characteristics, swelling behavior, and bioactive substances’ release performance. Bacterial cellulose (BC), a non-toxic, biodegradable polymer of renewable origin, has been used to develop hydrogels with IPNs via the radical copolymerization of acrylic acid (AA), using N, N-methylene-bis-acrylamide (MBA) as the crosslinker. *Hypericum perforatum* L. and *Melissa officinalis* L. ethanolic hydroalcoholic phytoextract 70% was originally encapsulated directly into the IPN hydrogels during synthesis via a less complicated and safer route for soil application. The novelty arose from the use of EHP as a loading medium within an IPN hydrogel matrix for the development of slow-release agrochemical formulations. *Hypericum perforatum* L. *(St. John’s Wort)* and *Melissa officinalis* L. have attracted significant attention due to their high contents of phenolic compounds (flavonoids and tannins) and strong properties, including antifungal, antimycobacterial, antioxidant, and antiviral activities [38,39], indicating their potential for the development of biopesticides and biofungicides, or bioinsecticides [40].

According to the literature [41,42,43], the combination of a biopolymer with a synthetic polymer improves biodegradability, mechanical properties, and plant growth performance, while also reducing production costs. This is one of the reasons why the IPN hydrogels proposed in the study use BC, AA, and MBA, available and inexpensive raw materials, and a low energy-consuming production process, i.e., free radical polymerization at 30 °C. In addition to economic advantages, another objective of the study is to improve the mechanical properties of the novel IPN hydrogels and ensure that they meet the level of rigor required for the intended application. In this respect, the physicochemical and structural properties of the IPN BC-poly(AA-co-MBA) hydrogels were optimized for water adsorption capacity and mechanical strength. Furthermore, the performance of the developed hydrogels was evaluated through controlled-release studies in simulated aqueous environments, highlighting their potential as natural and eco-friendly carrier systems for plant-derived bioactive compounds. By applying multiple kinetic models (zero-order, first-order, Higuchi, Korsmeyer–Peppas, and pseudo-second-order), we elucidate the mechanistic pathways governing the release of bioactive compounds and demonstrate how synthesis parameters influence both the physical properties and potential agricultural functionality of the hydrogels.

## 2. Results and Discussions

### 2.1. Rheological Properties of the IPN BC-Based Hydrogels

**Figure 1:**
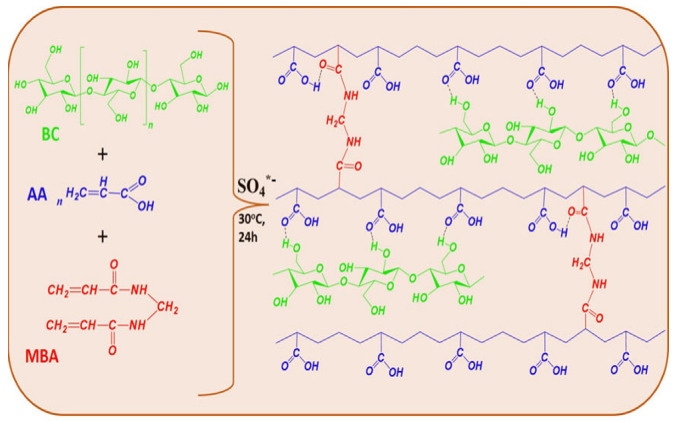
Proposed interpenetration mechanism between BC and the crosslinked poly(AA-co-MBA).

In order to follow the rheological behavior of the prepared hydrogel, it is highly important to understand the interpenetration mechanism between BC and the crosslinked poly(AA-co-MBA) [14]. In this respect, it is presumed that two types of interactions influence the formation of IPN hydrogels, as presented in Figure 1. First, the formation of hydrogen bonding between the (-OH) groups in the BC backbone and the (-COOH) groups in PAA, and second, the interaction between the carboxylic (-COOH) groups in PAA and the amide (-CONH-) groups in MBA. Thus, the increase in the BC content should have a positive effect upon the mechanical strength of the final gels.

**Figure 2:**
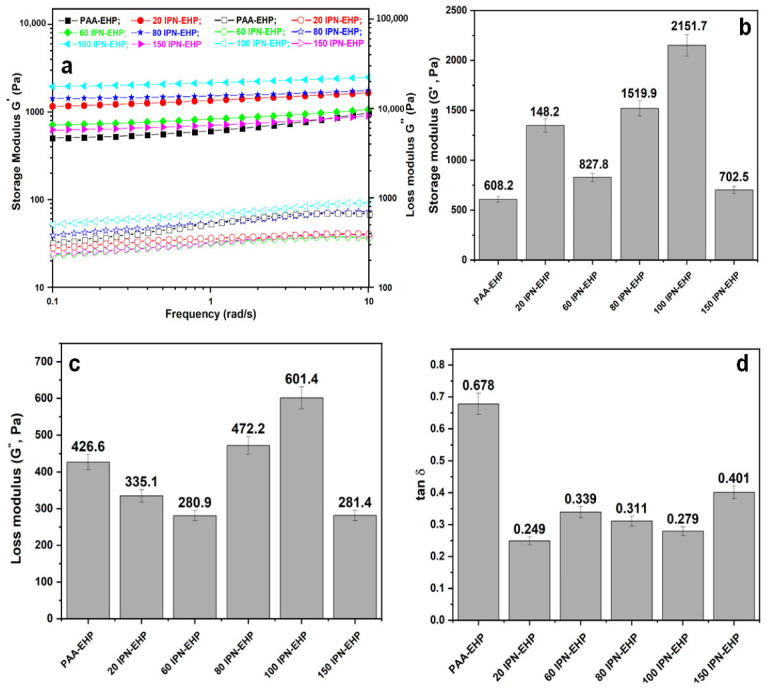
Rheological properties of the IPN hydrogels. Frequency dependence of the storage modulus (G′) and loss modulus (G″) for the IPN hydrogels (**a**). Storage modulus (G′) at 1 Hz (**b**), loss modulus (G″) at 1 Hz (**c**), and tan δ at 1 Hz (**d**) for the BC-based IPN hydrogels.

Furthermore, to highlight the influence of the BC content, a frequency-dependent shear test was performed to evaluate the rheological properties of IPN hydrogels swollen at equilibrium. The results are graphically represented in Figure 2. The storage modulus (G′) can be considered an indicator of the mechanical strength of the hydrogels [44,45]. For this reason, the storage (G′) and loss (G″) moduli were measured over a frequency range of 0.1–10 Hz to assess the viscoelastic behavior of the IPN hydrogels (Figure 2a). All IPN hydrogels exhibited solid-like rheological behavior, as evidenced by the storage modulus (G′) being consistently higher than the loss modulus (G″) values. Higher G′ values indicate a greater degree of elastic behavior relative to the viscous (viscoelastic) response [46]. It can also be observed that the slow increase in G′ values with frequency, likely due to enhanced interactions between BC fibers and PAA chains within the IPN matrix, indicates a slow relaxation process characteristic of rigid materials, which means the results are promising for the development of IPN hydrogel materials with enhanced mechanical strength. Additionally, the values of G′ (Figure 2b), G″ (Figure 2c), and tan δ (Figure 2d) were compared at 1 Hz. Figure 2b shows that incorporating BC increases the mechanical stiffness of the IPN hydrogel. In Figure 2b, a clear increase in the storage modulus (G′) was observed for IPN BC-based hydrogels (IPN-EHP) compared with the control hydrogels (PAA-EHP). As expected, for the IPN-EHP hydrogels synthesized with different BC concentrations, the storage modulus G′ increased with the increase in the BC content; the highest G′ value was obtained for the 100IPN-EHP formulation (100 wt. % BC relative to AA). In addition, the 100IPN-EHP formulation (Figure 2c) also exhibited the highest G″ values, indicating superior energy dissipation capabilities [45]. The loss tangent (tan δ) values for the IPN-EHP hydrogels suggest a more elastic nature compared with the control PAA-EHP hydrogels (0 wt% BC) (Figure 2d). A decrease in tan δ below 1 shows that the IPN hydrogel behaves as a predominantly elastic, solid-like material [47] and may indicate changes in the crosslinking network that enhance mechanical strength. This latter characteristic may be beneficial for applications requiring increased stiffness [48]. In conclusion, the addition of BC to the hydrogels significantly improved the mechanical stiffness and overall stability of the IPN hydrogels.

### 2.2. IPN Structure Analysis Using FTIR Spectroscopy

**Figure 3:**
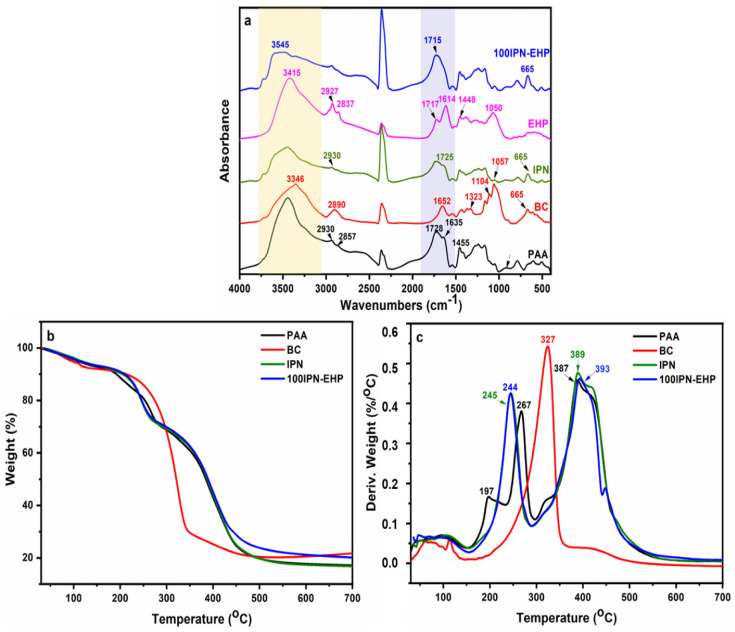
(**a**) FTIR spectra for PAA, BC, IPN, EHP, and 100IPN-EHP samples; (**b**) TGA and (**c**) DTG curves of PAA, BC, IPN, and 100IPN-EHP.

FTIR analysis was carried out to characterize the chemical structure of the materials and to evaluate the interactions occurring between the components of the IPN hydrogels. Figure 3a presents the FTIR spectra of the PAA-based xerogels, neat BC, the IPN xerogels (IPN), as well as the IPN xerogels prepared from BC and PAA in the presence of EHP (IPN EHP).

The infrared spectrum of the PAA-based xerogel displays the characteristic absorption bands of CH and CH_2_ groups at 2930 cm^−1^ and 2857 cm^−1^, respectively. The peak at 1728 cm^−1^ corresponds to the protonated carboxylate groups forming a cyclic dimer, while the two bands at approximately 1634 cm^−1^ and 1455 cm^−1^ are assigned to the asymmetric and symmetric stretching vibrations of the C-O bond in carboxyl groups, respectively. The broad absorption band in the 3100–3700 cm^−1^ region can be attributed to the O-H stretching vibrations of carboxylic acids in AA [49,50].

The characteristic spectrum of neat BC shows bands at 3346 cm^−1^ and 1652 cm^−1^, corresponding to the presence of a large number of hydroxyl (OH) groups, and a band at 2890 cm^−1^, attributed to the CH_2_ functional group. The presence of cellulose was confirmed at 1323 cm^−1^ (CH_2_ symmetric group), 1110 cm^−1^ (asymmetric vibration of the C-O-C glycosidic ring), 1050 cm^−1^ (vibration of the C-O group for primary and secondary alcohols), and 665 cm^−1^ (OH symmetric) [51,52,53].

The EHP spectrum shows the most intense band in the region 3600–3200 cm^−1^, characteristic of H-bonded vibrations of the polymeric hydroxyl group (-OH) in polyphenolic compounds (flavonoids, phenolic acids) [54]. Also, the bands at 2930 cm^−1^ and 2837 cm^−1^, characteristic of aliphatic C-H vibrations from CH_2_ groups, are also indicative of terpenoids and bitter principles. The bands at 1717 cm^−1^ and 1620 cm^−1^ are characteristic of the asymmetric and symmetric C=O vibrations of the ketones, esters, and carboxylic acids, characteristic of quinones, coumarins, and flavonoids. The absorption band at 1057 cm^−1^ corresponds to the C-O vibration, characteristic of primary and secondary alcohols [55].

Furthermore, the spectrum of IPN xerogels based on BC-poly(AA-co-MBA) and encapsulated EHP (100IPN-EHP) exhibited slightly shifted bands characteristic of PAA, such as the intense broad band with a maximum of 3545 cm^−1^, which was assigned to the hydroxyl (−OH) stretching vibration of AA units, overlapping with the hydroxyl (−OH) stretching vibration of the BC and O–H and characteristic of the polyphenolic of the EHP [14], and the band at 1715 cm^−1^, which was assigned to the protonated carboxylate groups of the AA units, overlapping with the C=O vibrations of the ketones, esters, and carboxylic acid of the EHP. Therefore, the IPN BC-poly(AA-co-MBA) hydrogels in the EHP medium were successfully synthesized through free radical polymerization.

### 2.3. Investigation of Thermal Behavior (TGA/DTG)

The decomposition and thermal stability of the fabricated xerogels were evaluated through the TGA technique, and the corresponding results are presented in Figure 3b,c. The TGA curve of the simple PAA-based hydrogel is more complex compared to BC and typically reveals a multi-stage thermal degradation process. The TGA curve of the PAA-based xerogel highlighted a total mass loss of 82.79%. The first stage, with a maximum decomposition temperature of 197 °C, corresponded to the dehydroxylation of the carboxylic acid groups (anhydride formation), accounting for approximately 15.85% of the total mass loss. This was followed by the main degradation step, which exhibited a maximum decomposition rate at 267 °C and a mass loss of 15.01%, associated with the decarboxylation of PAA. The final stage, with a maximum decomposition temperature of 387 °C, included the loss of short-chain fragments formed via the exothermic chain scission of the polymer [56], amounting to 51.94%. Meanwhile, BC degraded in a single step, with a maximum decomposition rate at 327 °C, which can be attributed to overlapping processes of depolymerization, dehydration, and the cracking of glucose units from the cellulose skeleton [57], resulting in a total mass loss of 86.62%.

The thermal degradation behavior of xerogels based on BC and PAA, obtained in distilled water (IPN) and in extract solution (100IPN-EHP), is, however, similar to that of PAA-based xerogels, which undergo two degradation stages corresponding to the dehydroxylation of the carboxylic acid groups and the decarboxylation of PAA, with the maximum values shifted to lower temperatures compared to the synthetic hydrogel. The first degradation step registered maximum rates at 245 °C (for IPN) and 244 °C (for IPN-EPH), with cumulated mass losses of 29.72% and 29.08%, respectively. Different from PAA, the IPN hydrogel presented a more stable polymer network due to interpenetration with BC, as indicated by the higher maximum decomposition temperature of the polymer backbone, registered at 389 °C. The 100IPN-EHP xerogel shows an even higher maximum decomposition temperature of 393 °C, further supporting the beneficial role of the EHP in reinforcing the hydrogel matrix and acting as a physical crosslinker that improves both the mechanical strength and stability of the hydrogel [58]. The polyphenols present in EHP (caffeic, ferulic, and coumaric acids) are known for their inhibitory effects and can influence free radical polymerization by scavenging free radicals. As components of the polymerization medium, they can modify solvent–polymer interactions and chain conformation during network formation [59]. As a result, changes in chain stretching may promote the formation of topological entanglements within the polymer network [60]. These entanglements act as physical crosslinks, restricting network expansion [61] and increasing the energy barrier to thermal decomposition, generally improving the material’s structural integrity [62] and, consequently, its thermal and mechanical behavior [63,64]. In addition, the TGA curves (Figure 3b) show a total weight loss of 83.92% and 79.89% for the IPN and 100IPN-EHP xerogels, respectively. The thermal decomposition of naphthodianthrones and polyphenols in the extract overlaps with the decomposition process of BC, occurring between 330 and 350 °C [65]. Overall, the thermal analysis indicates that the introduction of the EHP into the hydrogel matrix exerted a structurally stabilizing effect, thereby improving the thermal resistance of the IPN networks.

### 2.4. Investigation of IPNs’ Morphology Using Scanning Electron Microscopy (SEM)

**Figure 4:**
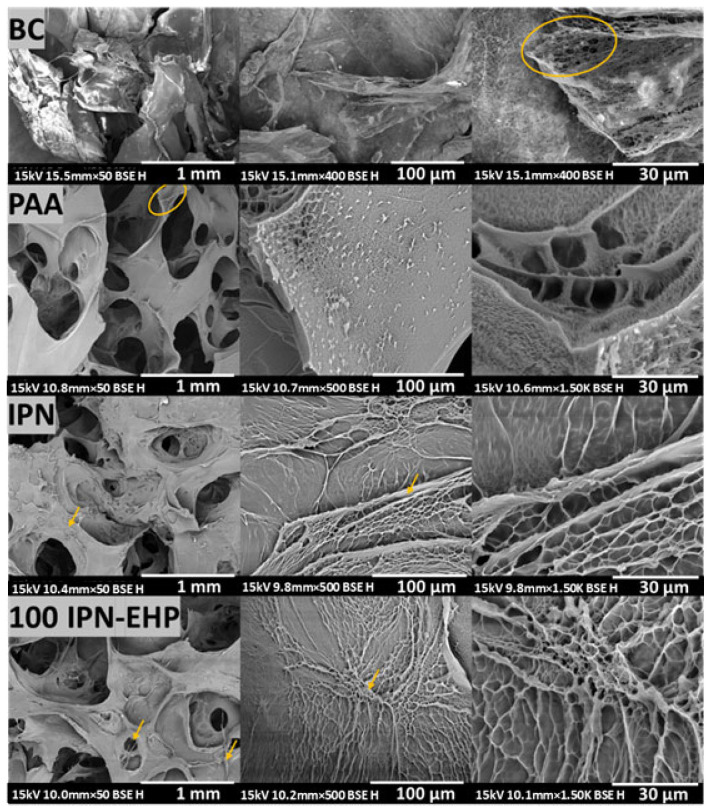
SEM images of freeze-dried control hydrogel and the IPN hydrogels at different magnifications.

SEM investigations revealed the morphological features of the freeze-dried control hydrogel and the IPN hydrogels (Figure 4—samples at different magnifications). The SEM images of the lyophilized BC exhibit a compact, mesh-like cellulose network composed of randomly oriented fibrils, corresponding to nanoscale fibers physically crosslinked with one another and forming a highly entangled structure with pores of various sizes distributed throughout the matrix [66].

The SEM images of the lyophilized PAA hydrogel show a homogeneous, microporous morphology characterized by opened and interconnected pores (100–1000 µm), along with a surface displaying curved, whisker-like structures that facilitate high levels of water adsorption [67]. Regarding the BC-poly(AA-co-MBA) IPN hydrogels (noted as IPN in Figure 4), the surfaces exhibited a porous morphology with interconnected pores, although both homogeneity and pore size were reduced compared with the neat PAA hydrogel. This behavior is probably due to AA’s strong interactions with the BC fibers during the impregnation stage, which leads to a more compact structure with confined space after polymerization/crosslinking [68]. Thus, the PAA network and BC chains became interpenetrated, confirming the interaction/polymerization mechanism responsible for the formation of the IPNs (see Figure 1). The SEM images also revealed the characteristic fibrous and multilayered arrangement of the lyophilized and milled BC. In the case of the IPN BC-poly(AA-co-MBA) hydrogels with EHP (noted as 100IPN-EHP), the samples exhibited a morphology similar to that of the control IPN hydrogels, forming swollen 3D network structures (Figure S1). Additionally, a quantitative analysis of the SEM images was performed using ImageJ software, V 1.54k, to provide a more objective evaluation of the IPN hydrogels’ morphology, and the corresponding data are presented in Table 1 and Figure S2. The pure PAA hydrogel exhibited the highest apparent porosity (46%), with an average pore area of 87,304 µm^2^ and an average pore diameter of 134.98 µm. In contrast, the IPN BC-poly(AA-co-MBA) hydrogels synthesized in an aqueous medium (100IPN) showed a substantially lower porosity (23.40%), accompanied by a reduction in average pore area (20,008 µm^2^) and average pore diameter (71.33 µm). For the IPN hydrogels synthesized in the EHP medium (100IPN-EHP), a porosity value of 28.67% was obtained, together with an average pore area of 28,607 µm^2^ and an average pore diameter of 84.36 µm. These quantitative results support the conclusion that the incorporation of BC leads to the formation of a denser polymer network with smaller pores, while the presence of EHP partially increases the porosity of the resulting IPN hydrogels. The slightly increased porosity may be attributed to the influence of ethanol on solvent–polymer interactions during polymerization and network formation. This structural configuration could facilitate multidirectional water diffusion while providing greater control over the release process, extending the slow-release duration and making these IPN hydrogels suitable carriers for the sustained delivery of the bioactive agent in agricultural applications [69].

### 2.5. Swelling Study of the IPN Hydrogels

**Figure 5:**
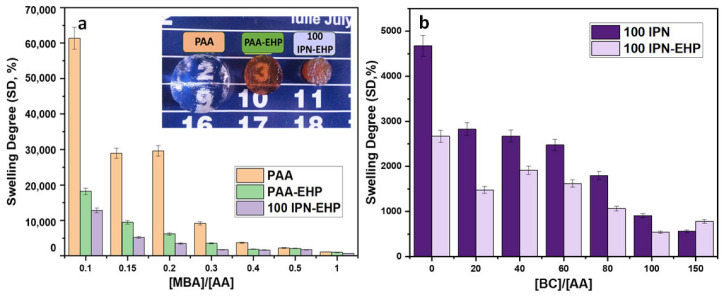
Influence of [MBA] concentration (**a**) and [BC] content (**b**) on swelling degree values for control hydrogel and IPN hydrogel samples.

To evaluate the influence of the MBA concentration on the SD (swelling degree, SD), the amount of crosslinker was varied while all other components, i.e., [AA] and the [KPS/MS]/[AA] ratio, were kept constant. As illustrated in Figure 5a, all synthesized samples exhibited similar swelling trends. An increase in the MBA content led to hydrogels with lower SD values. Reports in the literature indicate that higher crosslinker concentrations lead to a steady decrease in water absorption due to the formation of hydrogels with increased network density, reduced mesh size, and consequently diminished water absorption capacity [9]. It can also be noted that simple PAA hydrogels showed the highest swelling (Figure 5a, orange columns). When the polymerization medium was changed from distilled water to EHP solution, a significant decrease in the SD was observed (Figure 5a, green columns). This effect may result from the polyphenolic compounds present in EHP, which are known to exert inhibitory effects on the radical polymerization processes [61], thereby reducing the efficiency of network formation and ultimately lowering the swelling degree. The polyphenols present in EHP (e.g., caffeic, ferulic, and coumaric acids) may influence free radical polymerization due to their radical scavenging properties. In addition, when present in the polymerization medium, these compounds can alter solvent–polymer interactions and affect chain conformation during network formation. Padmavathi and Chatterji [62] reported that changes in chain stretching may promote the formation of topological entanglements within the polymer network. These entanglements can act as physical crosslinks, restricting network expansion and consequently influencing swelling behavior. Furthermore, the addition of BC, 1:1 relative to AA (Figure 5a, mauve columns), resulted in IPN hydrogels with significantly lower SDs vs. the corresponding systems without BC. These decreases in the SD can be attributed to the formation of new crosslinking bridges during IPN formation, leading to an IPN hydrogel with a higher crosslinking degree and, implicitly, lower SD values. Moreover, the IPN hydrogel with EHP (IPN-EHP) exhibited reduced transparency, indicating morphological and structural modifications arising from the interpenetration of BC fibers and within the AA polymer network, as illustrated in the inset of Figure 5, and supported by TGA and SEM analyses.

The influence of the BC concentration on the water absorption capacity was also investigated. As previously mentioned, six different BC concentrations were used to obtain IPN hydrogels with varying [BC]/[PAA] ratios, while all other components, i.e., [AA], [MBA]/[AA], and [KPS/MS]/[AA], remained constant. As illustrated in Figure 5b, increasing the BC concentration resulted in a progressive decrease in the swelling degree in both polymerization media (distilled water and EHP solution). This is not at all surprising because, as previously mentioned, the incorporation of BC into the hydrogel structure led to a decrease in the SD (Figure 5a). Therefore, a higher BC content increases the crosslinking density and the degree of chain entanglement, which restricts polymer network mobility, reduces the available free volume for water uptake, and slows the diffusion of bioactive molecules, ultimately resulting in a lower overall release rate. Conversely, a lower SD effectively decelerates the release of the bioactive molecule [70]. For agriculture applications, a lower SD is desirable, as it promotes the slower diffusion of the bioactive agent into water and enables a more controlled release profile [14]. Thus, by optimizing the slow-release behavior of the IPN hydrogel, precise bioactive agent delivery can be achieved, reducing the frequency and enhancing the utilization efficiency [71].

### 2.6. Controlled-Release Experiments for EHP-IPNs

**Figure 6:**
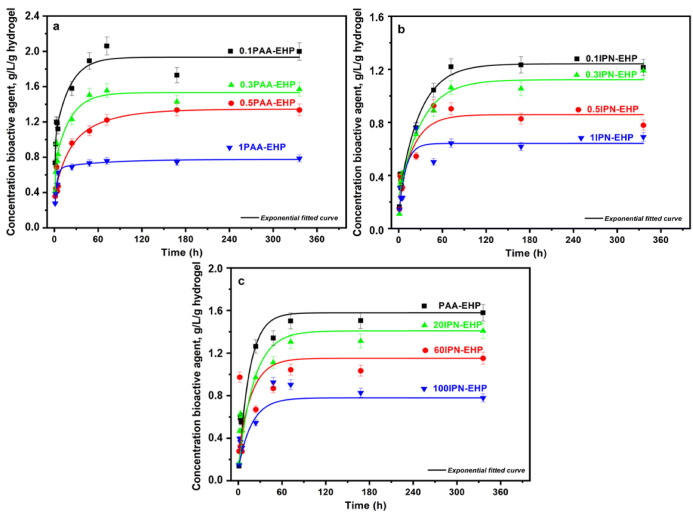
The release profiles of bioactive agents encapsulated in the matrix of neat PAA (**a**) and IPN hydrogels formulated with different MBA (**b**) and BC (**c**) concentrations.

The release behavior of IPN hydrogels is a key factor in assessing their suitability as carriers for various biologically active formulations, particularly within eco-friendly agricultural applications. The EHP was directly used as the reaction medium during hydrogel synthesis. Based on the extract concentration (0.6376 g mL^−1^) and the volume used during synthesis (2 mL), the maximum theoretical loading capacity of the xerogels was estimated to be approximately 21% (1.2752 g extract per 6 g dry xerogel), assuming complete retention of the phytoextract. Because the amount of extract potentially removed during the purification step was not quantified, this value represents a theoretical maximum rather than the actual loading efficiency. Accordingly, this study focused on evaluating the controlled-release performance of the EHP from hydrogels via UV-Vis analysis, considering systems prepared with different BC ratios and varying crosslinker concentrations in the extract-based polymerization media. Hydrogel systems prepared with different concentrations of the crosslinking agent, both neat PAA hydrogels and IPN structures synthesized in phytoextract-based media, are presented in Figure 6. Variations in the amount of MBA used during synthesis produced a clear and systematic influence on the release of the encapsulated bioactive compounds for both the neat hydrogels (Figure 6a) and the BC-containing IPN hydrogels (Figure 6b). As expected, increasing the density of crosslinking bridges resulted in a progressive reduction in the release rate, which is consistent with the formation of a more densely crosslinked polymeric network [72,73]. At the same time, the effect of incorporating BC into the hydrogel matrix becamee evident: the neat hydrogels released approximately twice as much bioactive agent as the corresponding IPN systems, highlighting the restrictive diffusion environment generated by the BC-reinforced network [27].

**Figure 7:**
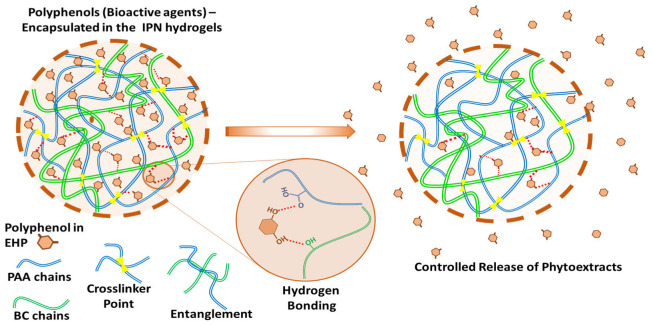
Design strategy for release profiles of IPN BC-poly (AA-co-MBA) hydrogels.

The controlled-release behavior of the phytoextracts encapsulated within the EHP-based systems, evaluated as a function of the BC concentration (Figure 6c), indicates that both the PAA-based neat hydrogel and the IPN composite hydrogels exhibited comparable release profiles. This outcome is consistent with expectations, as incorporating BC into the hydrophilic PAA matrix of the IPN structures resulted in a more compact polymeric network with reduced swelling capacity. According to the release behavior, the IPN hydrogels based on BC and PAA are likely reflecting the formation of hydrogen bonds and physical entanglements at the interface between BC fibers and PAA chains [73]. This is because the acrylic acid monomers were polymerized after penetrating the BC fibers, thereby facilitating the formation of physical entanglements between the two components, which may lead to the slow release of the encapsulated bioactive agents (Figure 7). Consequently, the systems displayed a sustained-release pattern characterized by an initial rapid discharge of bioactive compounds during the first 5 h, followed by a noticeably slower, continuous release extending up to 336 h. In agreement with previous reports, the release rate of the bioactive agents decreased as the BC content increased, with the most pronounced effect observed for the 100IPN-EHP formulation [14]. Thus, increasing the BC concentration may increase crosslinking by forming stronger physical entanglements, leading to a decrease in the amount of bioactive agent slowly released over time.

Strong non-covalent interactions may form between the IPN hydrogel matrix and polyphenol molecules from EHP, thereby trapping the bioactive compounds within the network and slowing their release [74]. A high concentration of phenolic hydroxyl groups enables the formation of numerous intermolecular hydrogen bonds with the hydroxyl groups of the IPN polymer matrix, which can contribute to a slow-release effect [75]. Thus, the bioactive agents in EHP, once encapsulated within the IPN structure and associated with the polymer chains, become embedded in the hydrogel and follow a more complex diffusion pathway, leading to sustained release (Figure 7) [76]. These results suggest that the IPN BC-PAA hydrogel has the potential to serve as a multifunctional material that enhances absorbent properties while simultaneously acting as a smart carrier for plant-derived bioactive compounds. Such characteristics may be advantageous for agricultural applications; however, further investigations are needed to assess their performance under realistic agricultural conditions [77].

To study the release kinetics, the zero-order, first-order, Higuchi, and Korsmeyer–Peppas models were applied to analyze the release profiles of IPN hydrogels. The fitted parameters (k, R^2^, r, and n) are summarized in Table S1. Among the evaluated models, the Higuchi and Korsmeyer–Peppas models provided the best fit to the experimental data, as indicated by their higher correlation coefficients (R^2^ = 0.98–0.99) and the greater linearity of the corresponding plots (Table S1). Graphical representations of all fitted models are provided in the Supplementary Data (Figures S3–S5). These results indicate that the release process is predominantly diffusion controlled. For the Korsmeyer–Peppas model, the fit was performed within its validity range (Mt/M∞ ≤ 0.6), ensuring the reliability of the extracted parameters. The incorporation of BC increased the crosslink density and entanglement of the polymer chains, thereby restricting network mobility and decreasing the diffusion rate of bioactive compounds [44]. The diffusion exponent (n = 0.20–0.40) was below the critical value of 0.45 for cylindrical geometry, indicating Fickian diffusion as the dominant release mechanism during the initial release stage. This interpretation is supported by the porous morphology observed via SEM and the experimental release profiles [78,79]. However, due to the extended-release period (up to 336 h), the contributions of polymer relaxation and swelling in the later stages cannot be completely ruled out.

**Figure 8:**
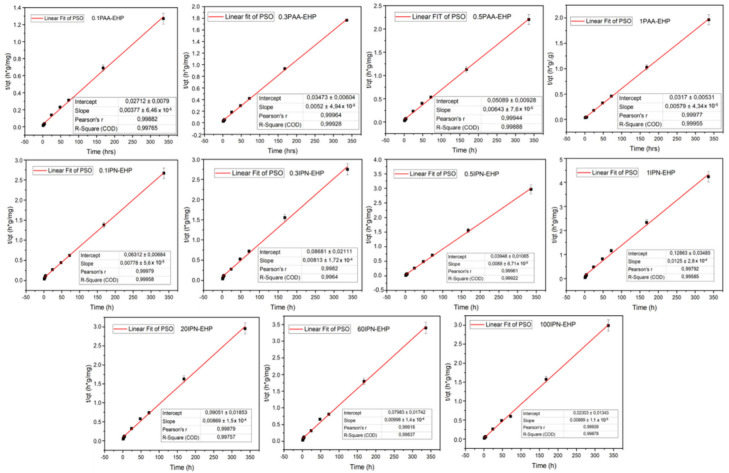
Linear fitting of pseudo-second-order (PSO) kinetic models used in the bioactive agents’ release from IPN hydrogels.

To further describe the release mechanism, the pseudo-second-order (PSO) model was also applied. Among all the models evaluated, the PSO model provided the best fit, with a correlation coefficient of R^2^ = 0.999 and the lowest relative error (Δq) (Table 2, Figure 8). Experimental data suggest that the release process is controlled not only by diffusion through the hydrogel network, but also by specific interactions between the bioactive compounds from EHP and the polymer matrix. In particular, polyphenols can form hydrogen bonds with the hydroxyl groups of the IPN hydrogel, delaying their release. Consequently, the mechanism can be described as a two-step process: (i) an initial faster release of the extract localized at the hydrogel surface, followed by (ii) a slower, sustained release controlled by the gradual disruption of the interactions, along with diffusion through the polymer network [80].

## 3. Conclusions

The main focus of this study was to develop a low-cost, environmentally compatible hydrogel with good adsorption capacity and favorable thermal and viscoelastic properties for potential agricultural use. Accordingly, this work reports the preparation of bacterial cellulose (BC) and poly(acrylic acid–co-N, N-methylene-bis-acrylamide) interpenetrating networks, which directly encapsulated a 70% ethanolic hydroalcoholic phytoextract (EHP) of *Hypericum perforatum* L. and *Melissa officinalis* L. during synthesis, as an integrated assessment of PAA–co–MBA hydrogels, examining how their structural features and swelling behavior influence the release of bioactive compounds. Thus, rheological data revealed a gradual increase in G′ with frequency, indicating strengthened BC–PAA interactions and slow relaxation behavior, typical of rigid systems, supporting the enhanced mechanical robustness of the developed IPN hydrogels. FTIR spectroscopy, TGA, and rheological measurements confirmed the structural organization and thermal resistance of the developed IPN BC-poly(AA-co-MBA) hydrogels in the EHP medium. SEM analysis showed a swollen 3D network with smaller but more numerous pores, a morphology that enhances multidirectional water diffusion and improves control over the release process, thereby prolonging the slow-release process. To assess the swelling behavior of the IPN hydrogels, the water uptake capacity was evaluated. Increasing the MBA or BC content led to higher crosslinking density and chain entanglement, limiting network mobility and reducing the free volume for water uptake. This restricted structure also slowed the diffusion of the bioactive agents, reducing the application frequency and enhancing the utilization efficiency.

In summary, the results demonstrate that the synthesis conditions directly shape the network architecture, which in turn governs both the water uptake capacity and release performance. Through this combined structural and functional analysis, the study provides a clearer understanding of the mechanisms that dictate the behavior of the hydrogels described herein, which appear to be suitable materials for agricultural applications and may ultimately serve as sustainable alternatives to fully synthetic acrylic-based absorbents. Further investigation into the topic, particularly regarding the materials’ performances in soil, should include field-scale evaluations to validate their behavior under real-life conditions, as well as the long-term monitoring of soil–IPN hydrogel interactions to confirm their ecotoxicity and stability. Moisture retention studies will also be addressed in a dedicated future investigation to provide a more comprehensive assessment of their water management capabilities.

## 4. Materials and Methods

### 4.1. Materials

The monomer acrylic acid (AA, 99%) purchased from Sigma-Aldrich (St. Louis, MO, USA) was distilled under a vacuum prior to use. N, N′-methylene-bis-acrylamide (MBA, 99%, Sigma-Aldrich, St. Louis, MO, USA) was used as crosslinker without any other purification. The aqueous ammonia solution (AA-NH^+^) (25%) was purchased from Chimopar SA (Bucharest, Romania). The redox system for radical polymerization, composed of potassium persulfate (KPS, 99%) and sodium metabisulfite (MS, 97%), was acquired from Acros Organics BV (Thermo Fisher Scientific, Geel, Belgium). Bacterial cellulose (BC) was obtained by collaborators at the National University of Science and Technology Politehnica Bucharest, Romania, produced in static culture using fructose as a carbon source and pollen as a nitrogen source [81], and delivered in acetic acid. *Hypericum perforatum* L. and *Melissa officinalis* L. ethanolic hydroalcoholic phytoextract 70% (EHP) was provided by the Commercial Society for Medicinal Plant Research and Processing PLANTAVOREL (Piatra Neamt, Romania). Moreover, 70% EHP refers to a hydroalcoholic phytoextract obtained using a solvent mixture composed of 70% ethanol and 30% water; the characteristics are presented in Table 3.

### 4.2. Synthesis of IPN BC-Poly(acrylic acid-co-N, N′-methylene-bis-acrylamide) Hydrogels in the Presence of EHP

**Figure 9:**
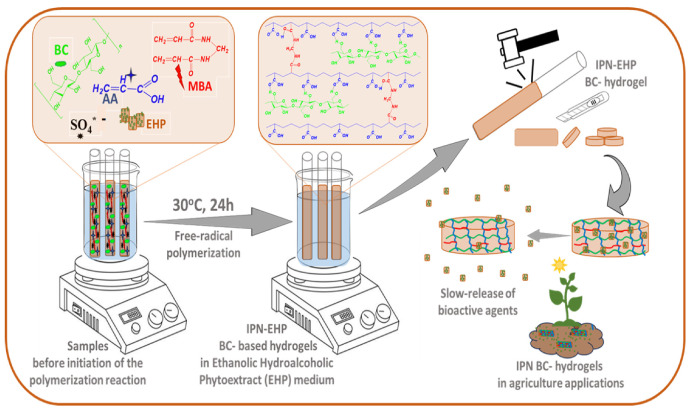
The synthesis process of the IPN BC-PAA-based hydrogels.

IPN hydrogels were synthesized via the radical copolymerization of acrylic acid with N, N′—methylene-bis-acrylamide (MBA). A redox system composed of potassium persulphate (KPS) and sodium metabisulphite (MS) was used to initiate the reaction in the presence of non-lyophilized bacterial cellulose (BC) in a 70% ethanolic hydroalcoholic phytoextract (EHP) medium (Figure 9). In the first step, the bacterial cellulose (BC) obtained from a static culture [82] was shredded to 1–2 mm particles in a blender for 10 min. Subsequently, the BC aqueous solution was filtered through filter paper for 2 h to reduce its water content to 40% of the total volume. In the second step, the media solutions corresponding to the redox initiation system (KPS and MS relative to [AA]) were prepared via mixing. AA (30% neutralized with NH3), MBA relative to [AA], and BC particles were introduced into 10 mm glass vials. Two sets of hydrogels were prepared: one containing EHP and the other containing only distilled water as a control sample. The resulting mixtures were subjected to ultrasonic treatment at room temperature in order to facilitate the adsorption of the monomers into the BC nanostructured network. After 3 h, the redox initiation system (KPS/MS) was added, the mixtures were ultrasonicated for homogenization (approximately 1 min), and then nitrogen gas (for about 5 min) was purged to remove oxygen. The vials were sealed using rubber septa and then placed in an oil bath to maintain the temperature of 30 °C for 24 h. At the end of the reaction, the vials were removed from the heating bath, cracked, and small disk-shaped pieces about 1 cm thick were cut from the resulting hydrogel rods in two.

Some of the hydrogel pieces were placed into an excess of distilled water for 5 days at room temperature for purification, changing the water daily in order to remove the unreacted monomer. The swollen hydrogels were then dried in an oven at 50 °C until they reached a constant mass, to determine the degree of swelling. This resulted in dry hydrogel disks (xerogels). The IPN hydrogels were denoted as either X IPN-EHP, where X represents the wt.% of non-lyophilized bacterial cellulose (BC) relative to AA, or IPN-EHP (Y MBA), where Y represents the wt.% of MBA crosslinker relative to AA. The composition of each hydrogel is shown in Table 4.

### 4.3. Determination of the Swelling Degree

The swelling degree (SD) of the control and IPN hydrogels was determined using an established methodology and equation, as described in the literature [9]:

(1) $$SD(g\;water/g\;dry\;polymer)=\frac{W_{h}-W_{x}}{W_{x}}\cdot 100$$

where W_h_ represents the weight at equilibrium of the swollen hydrogel, and W_x_ represents the weight of the xerogel (dried hydrogel).

### 4.4. The Release Tests of Bioactive Agents from Ethanolic Hydroalcoholic Phytoextract

The release measurements of bioactive agents from EHP in distilled water, by adjusting the medium pH to 7.4 and maintaining the temperature at 37 ± 0.5 °C, were performed in triplicate. Samples of 3 mL were withdrawn at each predetermined time interval and the UV-Vis absorbance at 312 nm was measured and correlated with the corresponding concentrations of bioactive agents from EHP. However, this method does not provide compound-specific information about individual phytochemicals. Figure S5 shows the calibration curve for bioactive agents from EHP used to calculate the concentrations released by the studied samples. The bioactive agent release patterns from the IPN hydrogels were analyzed by plotting the UV-Vis data and by identifying the most appropriate mathematical model for the release process. The mathematical models for substance release were as follows:

(i) Zero-order model:

(2) $$F=k_{0}\cdot t$$

(ii) First-order model:

(3) $$F=100\cdot (1-e^{-k_{1}\cdot t})$$

(iii) Simple Higuchi model:

(4) $$F=k_{H}\cdot t^{0.5}$$

(iv) Linear logarithm form of Korsmeyer–Peppas model:

(5) $$\;\log\frac{M_{t}}{M_{\infty }}=\log\left(k\right)+n\cdot log\left(t\right)$$

(v) Linear pseudo-second-order model:

(6) $$\frac{t}{q_{t}}=\frac{1}{k_{2}\cdot q_{e}^{2}}+\left(\frac{1}{q_{e}}\right)\cdot t$$

where *F* is the amount of bioactive agent released in time *t*, *k*_0_ is the zero-order release constant in units of concentration/time, *k*_1_ is the first-order release constant, *k_H_* is the Higuchi dissolution constant, *M_t_*/*M_∞_* is the fraction of bioactive agent released at time *t*, *k* is the kinetic constant characteristic of the bioactive agent–polymer system, *n* is the release coefficient, *q_t_* is the cumulative amount of bioactive agent released at time *t*, *q_e_* is the equilibrium release capacity, and *k_2_* is the pseudo-second-order kinetic constant.

### 4.5. Characterization Methods

The Fourier Transform Infrared Spectroscopy (FTIR) measurements were performed on a Bruker Vertex 70 instrument (Thermo Fisher Scientific, Waltham, MA, USA) using KBr pellets. The FTIR spectra were recorded in the range of 4000–400 cm^−1^, with 64 scans at a spectral resolution of 4 cm^−1^.

Thermal gravimetric analysis (TGA) was performed on a Thermal Analysis SDT600 instrument (TA Instruments, New Castle, DE, USA) by heating a sample of approx. 5 mg from room temperature to 700 °C with a heating rate of 10 °C/min under a nitrogen flow.

SEM images were recorded on the fracture surface of the previously swollen and freeze-dried hydrogel samples (2.5 Free Zone Labconco freeze dryer) using a TM4000 plus II tabletop instrument (Hitachi, Krefeld, Germany) at an acceleration voltage of 10 kV (image signal: BSE). The sample was first immobilized on a double-sided carbon tape and then coated with gold (~5 nm, using a Sputter Coater Q150R ES Plus -Quorum, Puslinch, Canada) to enhance conductivity. Using SEM pictures, ImageJ software V 1.54k allowed for assessing porosity quantitatively [83]. The porosity, average area, and average diameter of samples PAA, 100IPN, and 100IPN-EHP were extracted from SEM images by measuring around 200 individual pores.

The rheological measurements of the IPN hydrogels were performed using a HR 20 Discovery Hybrid Rheometer (TA Instruments, New Castle, DE, USA) with a 40 mm parallel plate geometry. The viscoelastic parameters were performed within the linear viscoelastic regime (LVR) to evaluate the storage (G′), loss (G″) moduli, and tan delta (δ). A shear stress of 1 Pa was selected, as it fell within this LVE range (Figure S6). Measurements were carried out as a function of angular frequency in the range of 0.1–100 Hz at 1% strain and a constant temperature of 25 °C.

The Ultraviolet-Visible Spectroscopy (UV-Vis) analysis was performed on a 500 Thermo Nicolet Evolution device (Thermo Electron Corporation, Cheshire, UK) by measuring the absorbance of the solution at λ = 312 nm; bioactive natural substances of phytoextracts (flavonoids, phenolic acids, and alkaloids) absorb at ~310 nm [82]. Based on the calibration curve, the amount of EHP released was determined (Figure S7). All measurements were performed in triplicate to ensure the accuracy and reproducibility of the results.

For statistical analysis, data were analyzed using Origin software (version 2018). One-way analysis of variance (ANOVA) was performed to compare individual results. A *p*-value < 0.05 was considered statistically significant. All experiments were conducted in triplicate, and the results are presented as mean ± standard deviation (SD).

## Figures and Tables

**Table 1:** Data obtained from ImageJ for simple hydrogel (PAA) and IPN hydrogels (100IPN and 100IPN-EHP).

Sample	Porosity (%)	Average Area (µm ^2^)	Average Diameter (µm)	Min. Diameter (µm)	Max. Diameter (µm)
PAA	46.10	87,304	134.98	22	2793
100IPN	23.40	20,008	71.33	22	1422
100IPN-EHP	28.67	28,607	84.36	20	1793

**Table 2:** Fitted parameters of pseudo-second-order (PSO) kinetic models used in the bioactive agents’ release from IPN hydrogels.

Sample	k_2_, (g/mg × h)	R^2^	$q_{e}$ (Calc.)	$q_{e}$ (Experim.) (mg/g)	Δq (%)
0.1PAA-EHP	0.00052	0.9977	265.25	268.32	−1.14
0.3PAA-EHP	0.00078	0.9993	192.31	192.85	−0.28
0.5PAA-EHP	0.00081	0.9989	155.52	154.58	0.61
1PAA-EHP	0.00106	0.9996	172.71	173.61	−0.52
0.1IPN-EHP	0.00096	0.9996	128.53	127.47	0.84
0.3IPN-EHP	0.00076	0.9964	123.00	123.55	−0.44
0.5IPN-EHP	0.00196	0.9992	113.64	115.38	−1.51
1IPN-EHP	0.00121	0.9959	80.00	80.38	−0.48
20IPN-EHP	0.00083	0.9976	115.07	114.84	0.21
60IPN-EHP	0.00125	0.9984	100.20	99.51	0.69
100IPN-EHP	0.00343	0.9988	112.49	114.48	−1.74

**Table 3:** Phytochemical characterization of *Hypericum perforatum* L. and *Melissa officinalis* L. ethanolic hydroalcoholic phytoextract 70%.

Characteristics	Ethanolic Hydroalcoholic Phytoextract (EHP)
pH	8.16
Density, g/mL	0.9079
Evaporation residue, wt. %	5.1
Extracted substances in ethanol 70% *v*/*v* wt.% from dried substance	56.1
Total flavonoid content expressed as rutin	0.6376 mg/mL of solution 12.5033 wt.% calculated or the dried substance
Total polyphenol content expressed as caffeic acid	0.4350 mg/mL of solution 8.5305 wt.% calculated for the dried substance
Total naphthodianthrones content expressed as hypericin	0.4350 g/g of solution 0.2045 wt.% calculated or the dried substance

**Table 4:** Composition of IPN BC hydrogels with various BC concentrations and MBA contents ^a^.

Sample Name	AA-NH+ (mol/L)	BC (% Rel. to AA)	EHP (mL, 6.4 µg/mL Dried Substance)	MBA (% M Rel. to AA)	KPS/MS (% M Rel. to AA)
PAA	6	-	-	0.5	1
PAA-EHP		-	2		
20IPN-EHP		20			
40IPN-EHP		40			
60IPN-EHP		60			
80IPN-EHP		80			
100IPN-EHP		100			
150IPN-EHP		150			
0.1IPN-EHP		100		0.1	
0.15IPN-EHP				0.15	
0.2IPN-EHP				0.2	
0.3IPN-EHP				0.3	
0.4IPN-EHP				0.4	
0.5IPN-EHP				0.5	

^a^ Samples without EHP were also prepared as controls and denoted as xIPN or yIPN, where x is wt.% of BC relative to AA, and y represents the wt.% of MBA crosslinker relative to AA.

## Data Availability

All data generated or analyzed during this study are included in this published article and its Supplementary Material. Further inquiries can be directed to the corresponding author. No large-scale sequencing datasets or external repository submissions were generated in this study.

## Supplementary Materials

The following supporting information can be downloaded at: https://www.mdpi.com/article/10.3390/gels12070624/s1, Figure S1: SEM micrographs of freeze-dried BC, control hydrogel based on PAA, the IPN hydrogels based on BC-PAA, and IPN-EHP based on BC-PAA-EHP, at different magnifications; Figure S2: ImageJ and presence of pores for PAA, 100IPN, and 100IPN-EHP; Figure S3: Fitted parameters and corresponding kinetic model plots used to evaluate the release behavior of bioactive agents from simple hydrogels PAA-EHP; Figure S4: Fitted parameters and corresponding kinetic model plots used to evaluate the release behavior of bioactive agents from IPN hydrogels; Figure S5: Fitted parameters and corresponding kinetic model plots used to evaluate the release behavior of bioactive agents from IPN hydrogels containing different BC concentrations; Table S1: Fitted parameters of the zero-order, first-order, Higuchi, and Korsmeyer–Peppas kinetic models used in the bioactive agents’ release from IPN hydrogels; Figure S6: Amplitude sweep measurement of the PAA hydrogel showing the linear viscoelastic (LVE) region used for selecting the applied shear stress; Figure S7: The calibration curve obtained using a standard EHP phytoextract solution with known concentrations.
