# Supplementary material for: Interpenetrating Polymer Networks Based on Bacterial Cellulose and Poly(acrylic acid–co-N, N-methylene-bis-acrylamide) as Carriers for Phytoextracts
Source: Gels. 2026 Jul 11;12(7):624. doi: 10.3390/gels12070624 (PMC13409546; doi:10.3390/gels12070624)
Supplement: Supplementary file 1 [file gels-12-00624-s001.zip › gels-4372687-supplementary.pdf]

# Supporting Information

for

# Interpenetrating Polymer Networks Based on Bacterial Cellu-lose and Poly(acrylic acid–co-N, N-methylene-bis-acrylamide) as Carriers for Phytoextracts

**Anamaria Zaharia †, Anita-Laura Chiriac †, Marinela-Victoria Iordanescu, Bianca Elena Stoica, Andrei Sarbu * and Tanta-Verona Iordache ***

Advanced Polymer Materials and Polymer Recycling Group, National Institute for Research & Development in Chemistry and Petrochemistry ICECHIM, Spl. Independentei 202, 6th District, 060021, Bucharest, Romania

* Correspondence: andr.sarbu@icechim.ro (A.S.); tanta-verona.iordache@icechim.ro (T.-V.I.)

† These authors contributed equally to this work.

## 1. SEM analyses

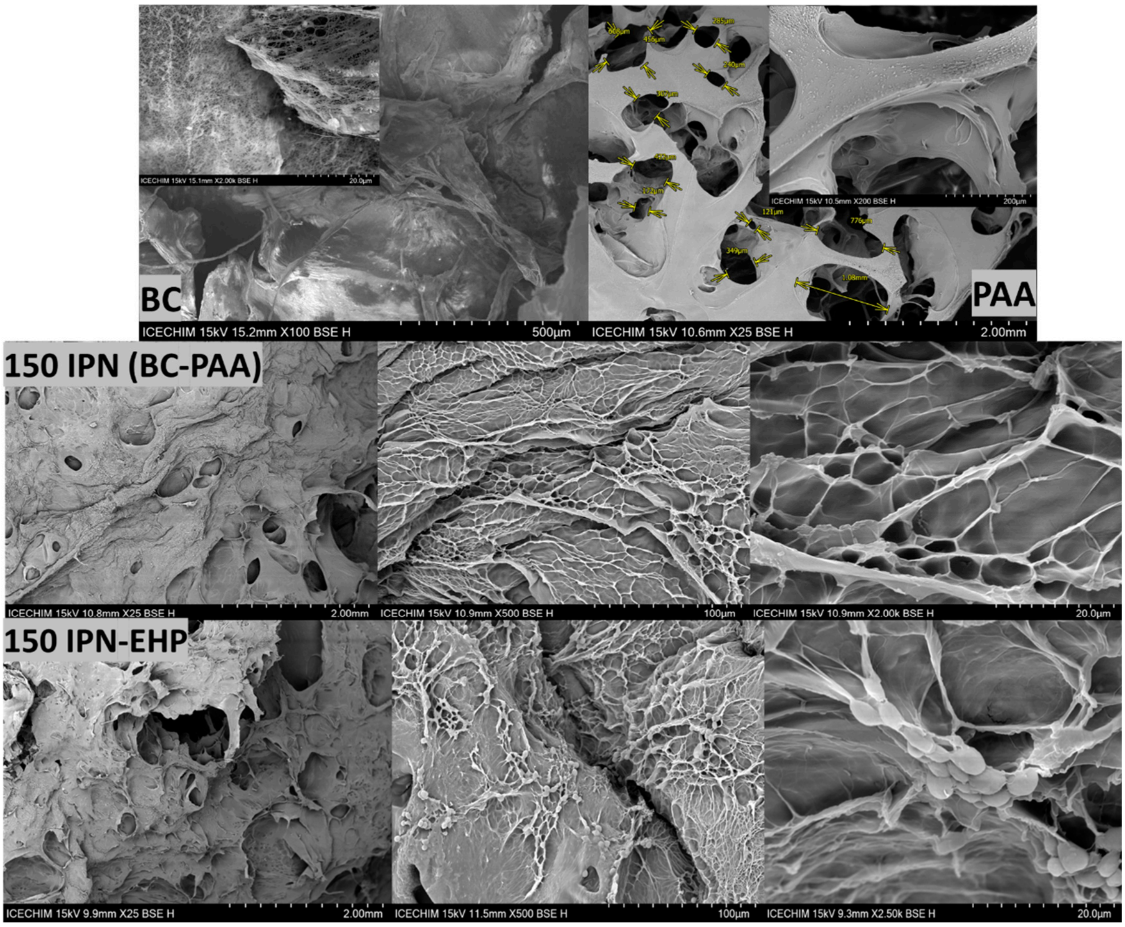

**Figure S1.** SEM micrographs of freeze-dried BC, control hydrogel based on PAA, the IPN hydrogels based on BC-PAA, and IPN-EHP based on BC-PAA-EHP, at different magnifications.

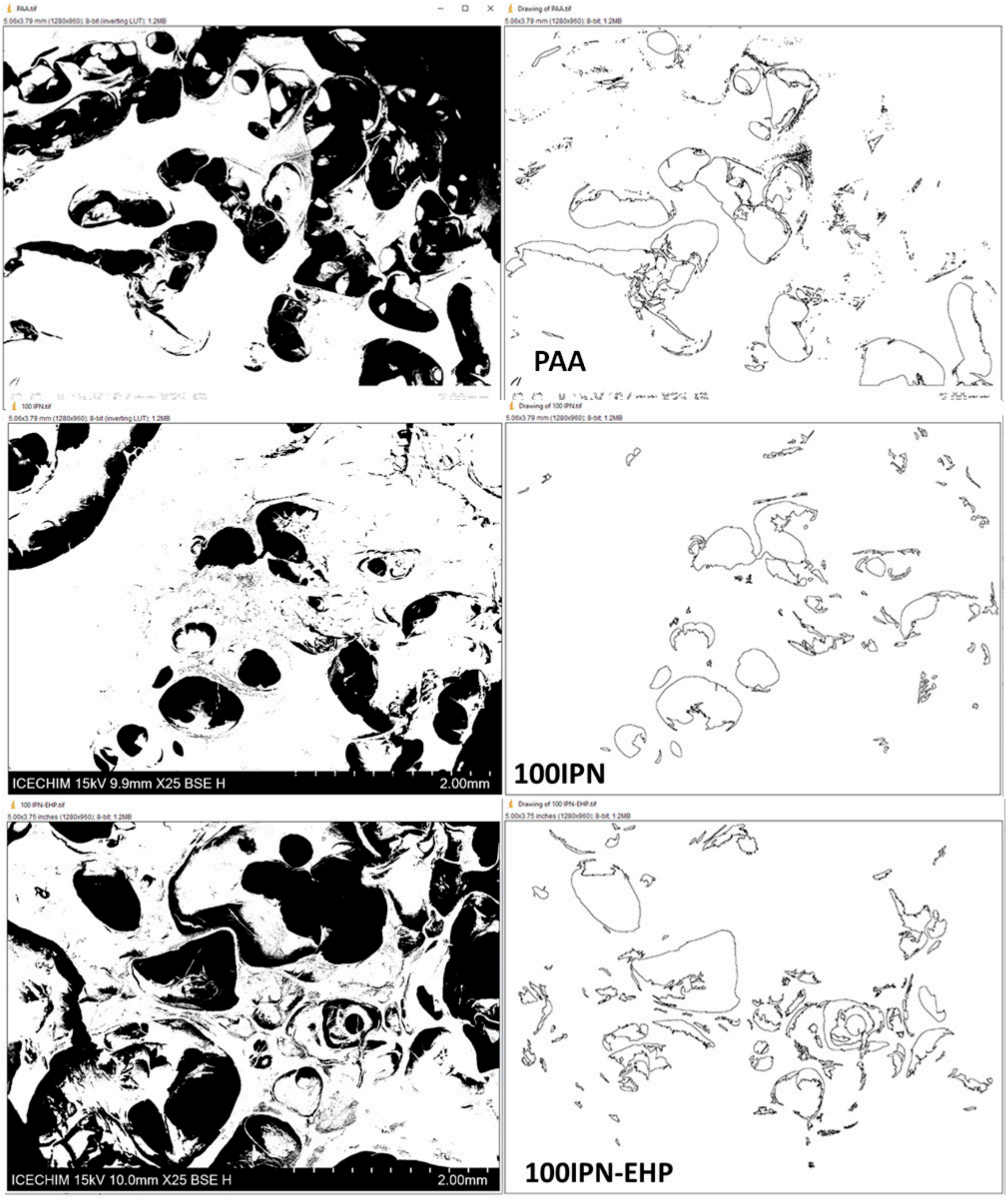


**Figure S2.** ImageJ and Presence of pores for PAA, 100IPN and 100IPN-EHP.

## 2. Supplementary release kinetics data were evaluated using the Zero-Order, First-Order, Higuchi, and Korsmeyer–Peppas models to characterize the release profiles of the IPN hydrogels.

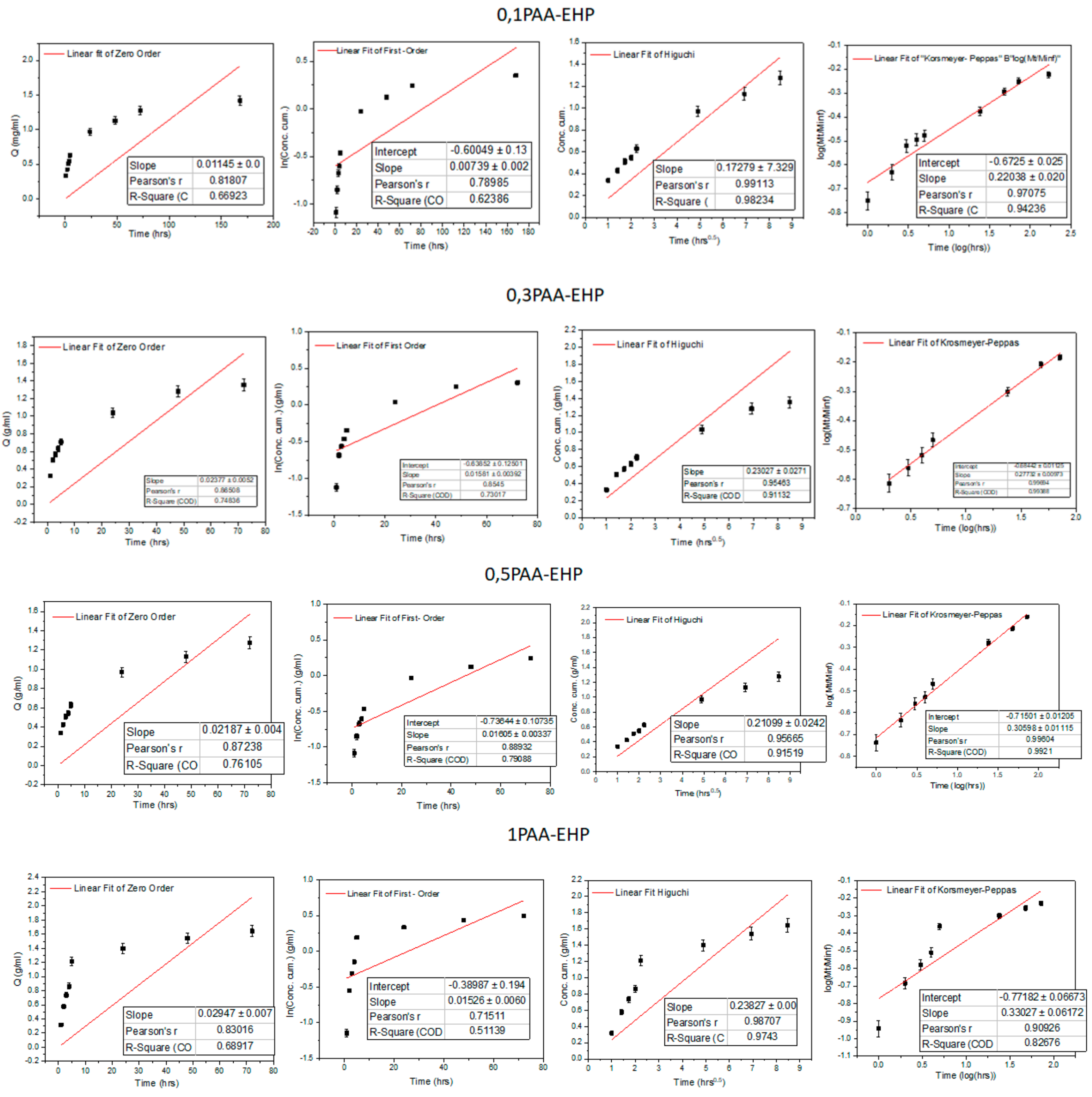


**Figure S3.** Fitted parameters and corresponding kinetic model plots used to evaluate the release behavior of bioactive agents from simple hydrogels PAA-EHP.

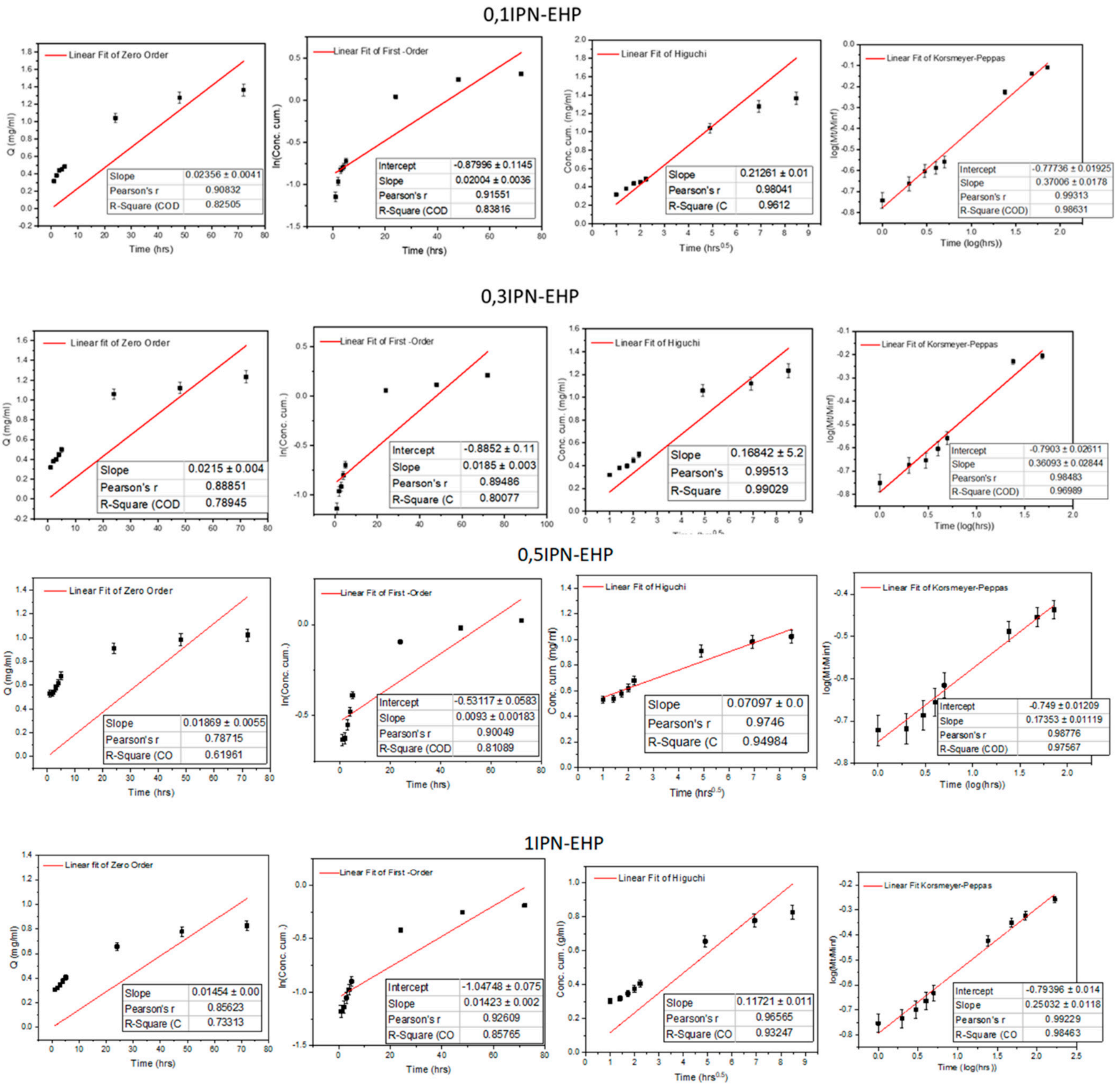


**Figure S4.** Fitted parameters and corresponding kinetic model plots used to evaluate the release behavior of bioactive agents from IPN hydrogels.

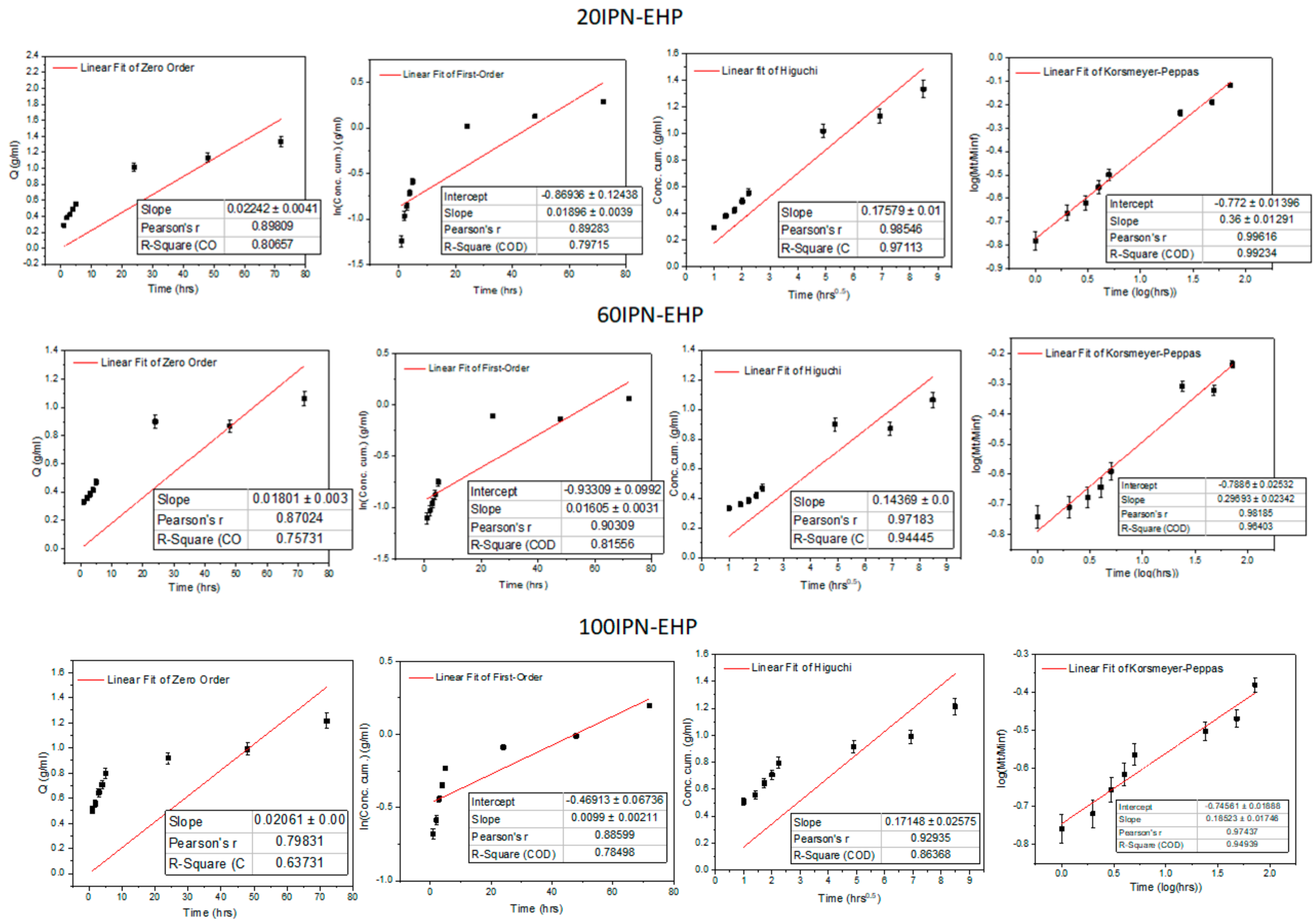


**Figure S5.** Fitted parameters and corresponding kinetic model plots were used to evaluate the release behavior of bioactive agents from IPN hydrogels containing different BC concentrations.

**Table S1.** Fitted parameters of the Zero-Order, First-Order, Higuchi, and the Korsmeyer–Peppas kinetic models used in the bioactive agents release from IPN hydrogels.

| Sample | Zero-Order | | | First-Order | | | Higuchi | | | Korsmeyer–Peppas | | | |
|---|---|---|---|---|---|---|---|---|---|---|---|---|---|
| | $k_0$, mg/mL | $R^2$ | r | $k_1$, $min^{-1}$ | $R^2$ | r | $k_H$, mg/mL·$min^{-1/2}$ | $R^2$ | r | $K_{KP}$ | n | $R^2$ | r |
| 0.1PAA-EHP | 0.011 | 0.669 | 0.818 | 0.007 | 0.624 | 0.790 | 0.173 | **0.982** | 0.991 | 0.212 | 0.220 | **0.942** | 0.971 |
| 0.3PAA-EHP | 0.024 | 0.748 | 0.865 | 0.016 | 0.730 | 0.855 | 0.230 | **0.911** | 0.955 | 0.207 | 0.277 | **0.994** | 0.997 |
| 0.5PAA-EHP | 0.022 | 0.761 | 0.872 | 0.016 | 0.790 | 0.889 | 0.211 | **0.915** | 0.960 | 0.193 | 0.306 | **0.921** | 0.996 |
| 1PAA-EHP | 0.029 | 0.689 | 0.830 | 0.015 | 0.511 | 0.715 | 0.238 | **0.974** | 0.987 | 0.169 | 0.330 | **0.826** | 0.909 |
| 0.1IPN-EHP | 0.024 | 0.825 | 0.908 | 0.020 | 0.838 | 0.916 | 0.020 | **0.838** | 0.916 | 0.167 | 0.370 | **0.986** | 0.993 |
| 0.3IPN-EHP | 0.022 | 0.789 | 0.889 | 0.019 | 0.801 | 0.895 | 0.168 | **0.990** | 0.995 | 0.162 | 0.361 | **0.970** | 0.985 |
| 0.5IPN-EHP | 0.019 | 0.620 | 0.787 | 0.009 | 0.811 | 0.900 | 0.071 | **0.950** | 0.975 | 0.178 | 0.174 | **0.976** | 0.988 |
| 1IPN-EHP | 0.015 | 0.733 | 0.856 | 0.014 | 0.858 | 0.926 | 0.117 | **0.932** | 0.966 | 0.161 | 0.250 | **0.985** | 0.992 |
| 20IPN-EHP | 0.022 | 0.807 | 0.898 | 0.019 | 0.797 | 0.893 | 0.176 | **0.971** | 0.985 | 0.169 | 0.36 | **0.992** | 0.996 |
| 60IPN-EHP | 0.018 | 0.757 | 0.870 | 0.016 | 0.816 | 0.903 | 0.144 | **0.944** | 0.972 | 0.163 | 0.297 | **0.964** | 0.982 |
| 100IPN-EHP | 0.021 | 0.637 | 0.798 | 0.010 | 0.785 | 0.886 | 0.171 | **0.864** | 0.929 | 0.179 | 0.185 | **0.949** | 0.974 |

## 4. The rheological measurements

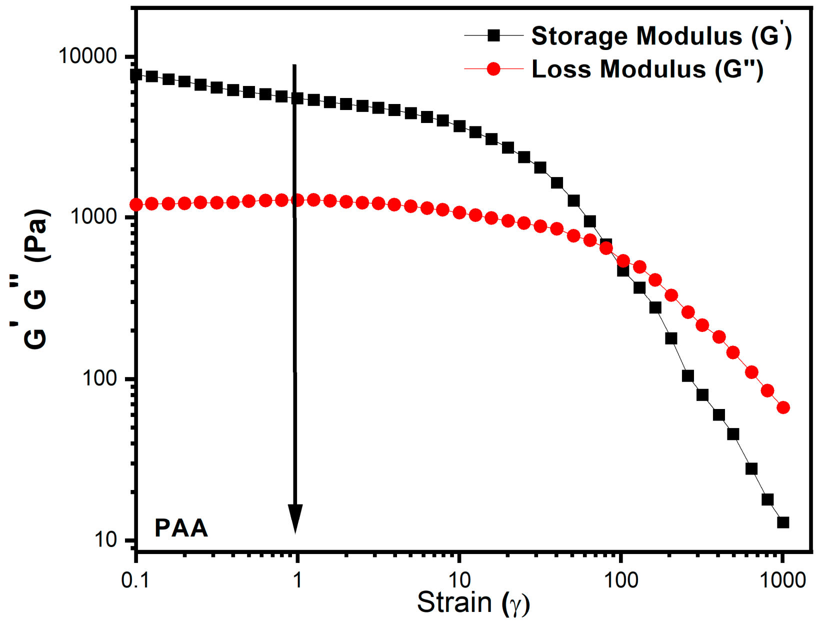


**Figure S6.** Amplitude sweep measurement of the PAA hydrogel showing the linear viscoelastic (LVE) region used for selecting the applied shear stress.

## 5. Determination of in vitro release of Hypericum perforatum L. and Melissa officinalis L. - Ethanolic Hydroalcoholic Phytoextract (EHP) from IPN hydrogels.

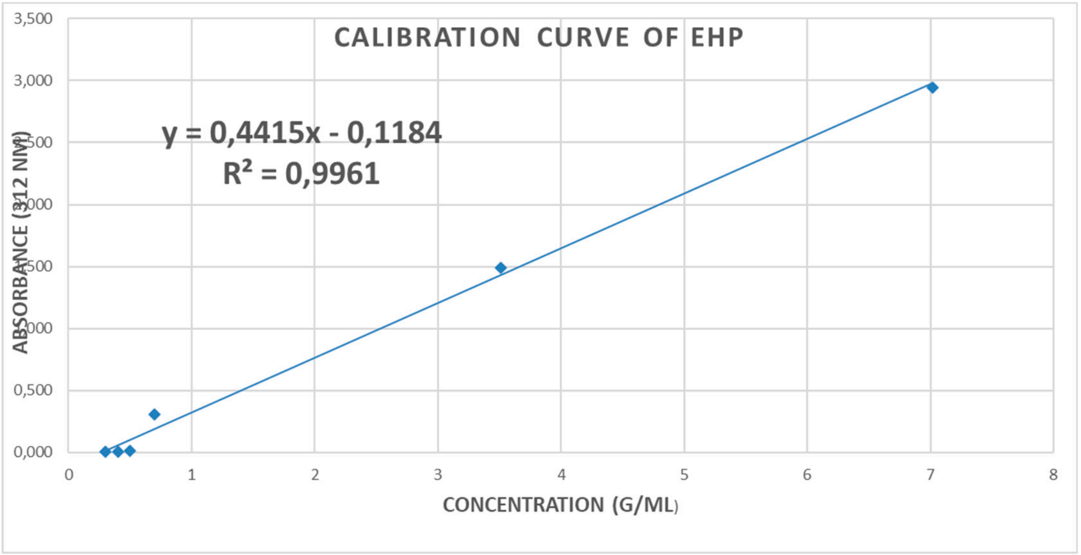


**Figure S7.** The calibration curve obtained using a standard EHP phytoextract solution with known concentrations.
